# The mechanism of action, clinical significance, and research progress of tumor antigen-specific T cells in the treatment of HCC

**DOI:** 10.3389/fonc.2026.1704905

**Published:** 2026-06-24

**Authors:** Liu Liu, Wang Tao, Lu Wei, Li Hongwei

**Affiliations:** Department of Radiation Oncology, The First Affiliated Hospital of Bengbu Medical University, Anhui, China

**Keywords:** combination therapy, hepatocellular carcinoma, immunotherapy, tumor antigen-specific T cells, Tumor microenvironment

## Abstract

Hepatocellular carcinoma (HCC) is a malignancy characterised by high incidence and mortality rates, with China accounting for over 50% of new cases and deaths globally. The prevention and treatment of HCC remain formidable challenges, primarily due to pronounced tumour heterogeneity and a complex immune microenvironment. Recent advances in immunotherapy have highlighted the critical role of tumour antigen-specific T cells (TASCs) in the management of HCC. This review systematically examines the mechanisms underlying TASCs function, recent advances in HCC research, and the clinical significance of TASCs detection. It further analyses current challenges in HCC immunotherapy and offers a theoretical foundation and prospective directions for the development of combination and personalised therapies targeting TASCs.

## 1 Introduction

HCC is the sixth most common malignant tumour globally and ranks fourth in cancer-related mortality, with incidence rates continuing to rise (1). In China, hepatitis B virus (HBV) and hepatitis C virus (HCV) are the predominant etiological factors (2). Compared to Western populations, Chinese patients with HCC are typically diagnosed at a younger age and at more advanced disease stages, resulting in higher tumour burden and accelerated disease progression. Recent advances in immunotherapy, such as the introduction of atezolizumab plus bevacizumab as first-line treatment for advanced HCC (3), have marked the onset of the immunotherapy era for this malignancy (4). Nevertheless, only 20-30% of patients derive sustained benefit from immune checkpoint inhibitors (ICIs), with the majority experiencing primary or acquired resistance (5). This underscores the incomplete understanding of TASCs regulation within the HCC immune microenvironment. TASCs denote T-cell clonal populations capable of recognising and targeting specific antigens expressed by hepatocellular carcinoma cells, serving as the core effector units directly executing tumour cell killing and achieving disease control (6). Therefore, the quantity, quality, and clonal diversity of TASCs are critical determinants of immunotherapy efficacy and patient prognosis (7). The tumour microenvironment imposes systematic suppression of TASCs at multiple levels, including impaired activation, defective tumour homing, profound functional exhaustion, and inadequate immune memory formation. While current immunotherapies seek to enhance TASCs function, their mechanisms, benefits, and limitations differ substantially, and a comprehensive comparison in HCC is lacking. It is important to note that, given chronic HBV infection as the primary aetiology of HCC in China, viral antigens play a central role in driving the carcinogenic process and subsequent antitumour immune responses. Therefore, in this review, to facilitate a focused and in-depth discussion of T-cell immunity in the context of virus-associated HCC, we have purposefully expanded the definition of TASCs: it encompasses not only T cells recognising classical tumour antigens, but also specifically includes T cells recognising HBV antigens. The latter also exert crucial effector functions in immune surveillance and immunotherapy responses within HBV-associated HCC. This review aims to provide a critical analysis of the stepwise suppression of TASC function by the HCC immune microenvironment, systematically comparing the rationale, advantages, and challenges of various TASC-targeted immunotherapies. Drawing on recent research, a combination therapy framework is proposed to establish a robust theoretical basis and guide the development of next-generation, more effective treatment therapies.

## 2 Hierarchical suppression of TASC function by the HCC immune microenvironment: impairment across the entire process from generation to dysfunction

**Figure 1:**
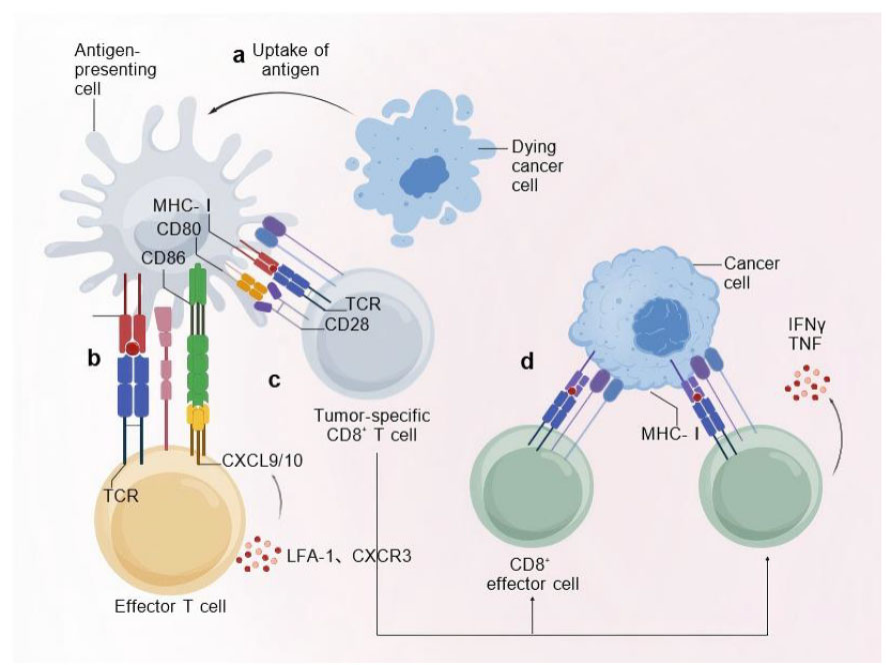
Positive feedback amplification mechanism in anti-tumour immunity **(a)** Circular starting point: Antigens released upon tumour cell death. **(b)** Central step: APC uptake, antigen presentation and reactivation of tumour-specific CD8+ T cells. **(c)** Effect execution: Activated T cells differentiate into effector cells, are chemotactically recruited to the tumour site, recognise and eliminate tumour cells. **(d)** Closure: Newly generated dead cells continue to provide antigens, sustaining this cycle and thereby amplifying the anti-tumour immune response.

The activation and clonal expansion of T cells require three distinct signals from antigen-presenting cells (APCs), as illustrated in **Figure 1**. The anti-tumour immune response is a complex, multi-step process (8–10). In HCC, TASCs are essential for tumour clearance; however, their activation and function are systematically inhibited by the tumour immune microenvironment. This suppression affects critical stages of the TASCs life cycle, thereby creating a multilayered regulatory network. A comprehensive understanding of this hierarchical suppression is crucial for the development of effective intervention strategies targeting these mechanisms.

### 2.1 Primary suppression: defective antigen presentation and failure of initial activation

The activation of TASCs begins when the T-cell receptor (TCR) specifically recognises the Major Histocompatibility Complex (MHC) on Antigen-Presenting Cells (APCs). T-cell activation, including TASCs, begins with recognition of the MHC-antigen peptide complex on APCs by the TCR (11). In the HCC microenvironment, this step is severely disrupted. Tumour-associated macrophages and myeloid-derived suppressor cells block APC maturation and function by releasing suppressive factors such as interleukin-10 (IL-10) and transforming growth factor-β (TGF-β) (12). This prevents APCs from migrating to lymph nodes and from effectively activating naive T cells (13). Regulatory T cells (Tregs) in the tumour exhibit high expression of cytotoxic T lymphocyte-associated protein 4 (CTLA-4). CTLA-4 binds to CD80/CD86 molecules on APCs more strongly than the co-stimulatory molecule CD28 does. This denies T cells the second signal they need for activation (14). Without co-stimulatory signals, clonal expansion and TASCs function are blocked, leading to dysfunction and exhaustion.

### 2.2 Physical and chemical barriers: vascular abnormalities and chemokine dysregulation impede T cell infiltration

Even when a small number of TASCs are successfully activated, the vascular system within HCC exhibits significant structural and functional abnormalities. These alterations not only induce tumour hypoxia and acidosis, creating an inhibitory metabolic microenvironment, but also directly impede T-cell adhesion and extravasation by modifying endothelial cell phenotypes and adhesion molecule expression. This physically restricts the infiltration of effector immune cells (15). Hepatocellular carcinoma cells exhibit high fatty acid synthase expression, leading to fatty acid accumulation in the tumour microenvironment that inhibits mitochondrial metabolism and function in TASCs. Concurrently, elevated expression of indoleamine 2,3-dioxygenase in the tumour microenvironment causes tryptophan depletion and kynurenine production, thereby inducing TASCs apoptosis or promoting their exhaustion (16). Concurrently, hepatocellular carcinoma cells and tumour-associated fibroblasts collectively form a chemical barrier by downregulating key T-cell chemotactic factors (e.g., CXCL9, CXCL10) and upregulating certain inhibitory chemokines. This prevents effector TASCs from infiltrating the tumour stroma, establishing the so-called “immune exclusion” or “immune desert” phenotype (17).

### 2.3 Functional suppression: multifaceted inhibitory signals induce profound T-cell exhaustion

TASCs that successfully infiltrate the tumour stroma remain strongly suppressed in their effector function by the HCC microenvironment, constituting a core component of HCC immune evasion (13). Key mechanisms include: under sustained antigenic stimulation, TASCs highly express multiple inhibitory receptors, such as programmed death-1 (PD-1), T cell immunoglobulin and mucin domain 3 (TIM-3), lymphocyte activation gene 3 (LAG-3), and T cell immunoglobulin and ITIM domain (TIGIT). Their corresponding ligands (e.g., PD-L1) are highly expressed on tumour cells and myeloid cells, transmitting potent negative regulatory signals that directly inhibit T cell proliferation, cytokine secretion, and cytotoxic function (18). Nutrient competition and metabolic waste accumulation driven by rapid tumour growth constitute further critical inhibitory factors: the CD39/CD73 extranucleotidase pathway converts extracellular ATP/ADP into immunomodulatory adenosine (19, 20). This adenosine directly suppresses T-cell function via the A2A receptor signalling pathway (21). Concurrently, high glycolysis in tumour cells and immunosuppressive cells generates substantial lactate, acidifying the microenvironment and further impairing T cell metabolic adaptability and effector function (22). Prolonged exposure to these multifaceted inhibitory pressures induces epigenetic remodelling in TASCs, stabilising their exhausted state. This condition proves difficult to fully reverse even upon removal of antigenic stimulation, representing a key mechanism underpinning resistance to immune checkpoint inhibitors. Notably, not all tumour-infiltrating T cells reside in a terminal exhausted state. This observation suggests the presence of a functionally plastic T cell subpopulation within the HCC microenvironment, potentially representing a target for reactivation by immunotherapy.

### 2.4 Impaired memory formation: long-term compromised immune surveillance

Effective anti-tumour immune responses should ultimately establish long-lasting immune memory to prevent tumour recurrence. However, during HCC progression, the generation of functional memory T cells is frequently disrupted. Due to insufficient initial T-cell activation and severe functional impairment during the effector phase, very few TASCs successfully differentiate into fully functional memory T cells. Notably, the generation of stem-like memory T cells (Tscm) and central memory T cells (Tcm)—which possess self-renewal and rapid response potential—is significantly reduced (23). This leads to weakened immune surveillance against tumours, thereby promoting tumour recurrence and metastasis.

## 2 Comparative and synergistic potential of TASCs in different immunotherapy strategies for HCC

TASCs play a pivotal role in controlling HCC progression through mechanisms such as regulating tumour growth and preventing recurrence (24). Recent clinical studies have demonstrated that TASCs activity is closely correlated with patient clinical outcomes across various immunotherapy approaches for different types of hepatocellular carcinoma. Immune checkpoint inhibitors (e.g., Anti-PD-1, Anti-PD-L1, Anti-CTLA-4 antibodies) (25–27), tumour-associated antigen peptide vaccines (e.g., MRP3, GPC3, AFP peptide vaccines) (28, 29), DC or adoptive T-cell therapy, the degree of enhanced TASCs activity (specifically CD8^+^T cells) following immunotherapy correlates significantly and positively with prolonged recurrence-free survival (RFS), and overall survival (OS), as well as reduced tumour recurrence rates. Moreover, the immune response to TASCs in hepatocellular carcinoma patients has significant clinical, pathological, and prognostic implications (30, 31). Recent reports indicate that TASCs responses to MAGE-A1, MAGE-A3, NY-ESO-1, SSX2, and SALL4 progressively decline from early to advanced HCC stages, potentially conferring immune protection in early disease. Conversely, TASCs responses, initially weak in early HCC, gradually increase in advanced stages, suggesting they may mark the malignant state of late-stage HCC (32). Therefore, dynamic monitoring of TASCs immune function in HCC patients not only provides clinicians with real-time, direct indicators for evaluating immunotherapy efficacy but also facilitates prognosis assessment following conventional treatments and early prediction of tumour recurrence.

## 3 TASCs as predictive and prognostic biomarkers for HCC: from basic research to clinical application

Dynamic monitoring of the reactivity of TASCs in HCC patients, specifically the HBV-specific TASCs defined in this review, holds significant value in guiding the clinical diagnosis and treatment of HCC, primarily manifested in three aspects: 1) Monitoring disease progression and determining prognostic outcomes: TASCs reactivity determines the course and outcome of infection (33). Patients with robust T-cell immune responses can clear the virus, whereas those with weaker responses develop chronic infection. TASCs gradually diminish and become depleted, ultimately progressing to cirrhosis and hepatocellular carcinoma (34). Thus, monitoring TASCs reactivity effectively tracks disease progression in patients with virus-associated HCC and provides crucial evidence for assessing prognosis.2) Evaluating treatment efficacy and guiding therapeutic strategies: During HCC treatment, changes in TASCs reactivity serve as a crucial indicator for assessing therapeutic outcomes. For instance, following nucleoside antiviral therapy in patients with virus-associated hepatocellular carcinoma, drugs effectively suppress viral replication, leading to a significant reduction in viral load and viral antigen levels. This subsequently results in a transient enhancement of TASCs’ immune responses within the patient’s body (35). Dynamic monitoring of TASCs’ reactivity enables timely assessment of treatment efficacy. Should post-treatment TASCs activity fail to markedly increase or even decline, this indicates potential treatment inadequacy, necessitating prompt therapeutic strategy adjustment to enhance outcomes.3) Predicting hepatocellular carcinoma outcomes and recurrence risk: TASCs reactivity is closely correlated with post-treatment outcomes and recurrence risk in hepatocellular carcinoma patients. Recent studies indicate that following surgical resection, prolonged recurrence-free survival in hepatocellular carcinoma patients is significantly correlated with the reactivity of TASCs (particularly CD8^+^T cells) in both liver tissue and peripheral blood (36). This finding suggests that monitoring the reactivity of TASCs in post-treatment patients can effectively predict disease outcomes and tumour recurrence risk, providing crucial guidance for clinicians in formulating postoperative follow-up strategies and intervention measures.

## 4 TASC-directed combined therapy for HCC

Given the pivotal role of TASCs in anti-HCC immunotherapy, all effective immunotherapeutic strategies ultimately seek, either directly or indirectly, to enhance and maintain TASCs populations. However, these approaches differ fundamentally in their mechanisms of action, intervention points, and associated challenges. Elucidating these distinctions is essential for the rational design of combination therapies.

### 4.1 ICIs: focusing on “reversal” rather than “creation”

ICIs, exemplified by PD-1/PD-L1 inhibitors, do not activate TASCs at their source. Instead, they aim to release TASCs that have infiltrated the tumour but remain functionally suppressed (37). However, their efficacy depends heavily on the presence of functionally salvageable T cells within the tumour. Research indicates that PD-1 antibodies primarily act on precursor exhausted cells within the exhausted T-cell lineage that retain some proliferative potential, exerting minimal effect on terminally exhausted cells whose epigenetic programmes are fully consolidated (38). This explains the limited monotherapy response rates of ICIs in HCC and points towards the need for combination therapies with other strategies that increase the number of TASCs or improve their functional state.

### 4.2 Cancer vaccines: aiming to initiate specific immune responses

Cancer vaccines aim to proactively induce the generation of novel TASCs targeting specific tumour antigens. Their success hinges on three key elements: antigenic components that enhance immunogenicity, effective delivery systems, and adjuvants overcome immune suppression. In HCC, multiple clinical studies have investigated vaccines targeting shared tumour-associated antigens such as AFP and GPC3. While demonstrating safety and the ability to induce immune responses, objective response rates remain low (29). The challenge lies in the low expression of these antigens in normal tissues, which can induce central tolerance and limit the generation of potent TASCs. Concurrently, the established immunosuppressive microenvironment rapidly suppresses emerging immune responses.

### 4.3 Adoptive cell therapy: reinfusion of selected or engineered TASCs

This strategy directly addresses several barriers to *in vivo* T cell activation. replenishing patients with T cells possessing anti-tumour activity. TIL therapy: Its advantage lies in the polyclonal nature of the reinfused T cells, which recognise patient-specific mutational neoantigens and, in theory, exhibit the strongest specificity (39). However, significant technical challenges exist in preparation: obtaining sufficient quantities of functional TILs from HCC tissue is inherently difficult, rapid *in vitro* expansion may exacerbate their exhausted phenotype, and cell persistence post-reinfusion remains a major challenge. CAR-T/TCR-T therapies: Through genetic engineering, T cells are equipped with navigation systems that specifically recognise tumour surface antigens (CAR-T) or MHC-presented intracellular antigens (TCR-T) (40). While achieving revolutionary success in haematological malignancies, these therapies face considerable difficulties in solid tumours such as HCC. Core obstacles include: 1) Target toxicity: e.g., GPC3 expression in normal liver tissue may cause hepatic injury; 2) Tumour heterogeneity: single-target approaches are prone to immune escape; 3) Complex immune microenvironment: engineered T cells face challenges of infiltration and suppression.

### 4.4 Radiotherapy: transforming cold tumours into hot tumours

Radiotherapy induces tumour antigen release, enhances antigen presentation, and alters the composition of immune cells within the tumour microenvironment (41). Combining radiotherapy with TASCs-directed immunotherapy promotes T-cell infiltration and functional restoration, proving particularly effective for locally advanced or immunologically “cold” tumours. No single treatment regimen is perfect. TASCs, as a plastic T-cell state, offer novel perspectives for combination therapies. Studies demonstrate that TASCs combined with PD-1 inhibitors and bevacizumab achieve an ORR of 32% and with a median progression-free survival (mPFS) of 8.6 months. This represents a significant improvement over the IMbrave50 regimen (ORR 27%, mPFS 6.8 months), with the mechanism attributed to bevacizumab enhancing vascular permeability and facilitating TASCs infiltration (3). TASCs represent a complex yet amenable critical node in tumour immunotherapy. A thorough understanding of their strong HLA restriction and TME sensitivity necessitates combination therapies to fully realize their potential.

## 5 Translational challenges and future directions for TASCs in HCC

Numerous HCC-associated tumour antigens have been identified, including AFP, GPC3, GP73, hTERT, MRP3, SART2, SART3, SALL4, and other tumour-associated antigens (32), cancer-testicular antigens such as MAGE-A1, MAGE-A3, NY-ESO-1, and SSX2 (42), and tumour neoantigens (36). However, very few T-cell epitope peptides from these tumour antigens have been reported in research studies. For instance, among CD8+ T-cell epitopes, only over 30 types exist for AFP, 16 for hTERT, and 11 for GPC3, with others being even fewer. Moreover, these are presented by only a handful of HLA-A molecules. Consequently, most researchers can currently utilise only a limited number of known epitope peptides to test a small subset of HCC patients carrying specific HLA molecules (43, 44). This approach fails to adequately reflect the functional state of the patient’s T-cell antigen-specific clonal repertoire (TASCs). Additionally, some researchers currently employ overlapping peptide libraries covering the entire length of tumour antigens (32, 45) or bioinformatically predicted epitope libraries (36) to test a broad cohort of HCC patients. Neither overlapping nor predicted peptide libraries contain functionally validated true epitopes; the vast majority are pseudoepitopes (28, 32, 36, 44), The T-cell responses elicited by these are markedly weaker than those induced by validated true epitopes. Furthermore, TASCs face persistent issues and challenges in HCC antitumour therapy. For instance, hepatocellular carcinoma exhibits high intratumoural heterogeneity, with only a minority of cells within the same tumour tissue highly expressing target antigens such as GPC3/AFP. This limits the efficacy of single-epitope-targeted TASCs in covering all tumour cells (46). Furthermore, the efficacy of ICIs varies considerably between patients, with some individuals potentially developing resistance. Regarding the relationship between immune checkpoint inhibitors and TASCs, some studies suggest that PD-1 inhibitors activate only certain subsets of exhausted T cells, while others propose that they comprehensively restore T cell function. CAR-T cell therapy and TIL therapy demonstrate potential for treating hepatocellular carcinoma, yet these techniques pose significant operational challenges, require stringent laboratory conditions, and incur high treatment costs, hindering widespread clinical adoption. This stems from the polymorphic nature of HLA molecules in human populations, the unclear antigenic peptide repertoire that triggers TASC responses, the scarcity of validated epitope peptides, and recent studies indicating that the vast majority of these peptides are pseudoepitopes (36, 47), severely compromising the authenticity of detection results. To address these challenges, future research must delve deeper into the mechanisms of TASCs in hepatocellular carcinoma, develop more effective immunotherapy strategies, and enhance treatment outcomes. Despite the numerous obstacles confronting TASC-based immunotherapy for HCC, ongoing investigations into its mechanisms are gradually transforming TASCs into tools with substantial clinical application potential, offering hope for overcoming current therapeutic limitations. Future research may focus on several avenues: the most immediate application lies in utilising them as dynamic predictive and prognostic biomarkers. As discussed herein, the intensity and quality of antigen-reactive TASCs demonstrate strong correlations with treatment efficacy across multiple therapeutic modalities. Consequently, continuous monitoring of these T cells in peripheral blood or tumour tissue could provide clinicians with a real-time, immunology-centric readout of treatment response, potentially surpassing or complementing traditional imaging-based assessments. This approach enables early identification of non-responders, allowing a timely transition to alternative therapeutic strategies.

In summary, whilst immunotherapy strategies centred on TASCs have demonstrated preliminary success, future breakthroughs will not lie in the simple addition of more drugs. Rather, they will require deep analysis of individual patients’ TASC status and tumour immune microenvironments to achieve precise matching and dynamic optimisation of treatment strategies. Where pre-existing T-cell responses are absent, strategies to initiate novel responses may be required. Consequently, analysing the tumour antigen TASC spectrum holds promise as a critical diagnostic tool for patient stratification and the customisation of combination immunotherapy regimens, thereby transcending current empirical approaches. The application of single-cell multi-omics technologies is enabling us to map unprecedentedly high-resolution ‘TASC landscapes’, providing a navigational framework for precision medicine. By performing single-cell RNA sequencing (scRNA-seq) and T-cell receptor sequencing (scTCR-seq) on tumour or peripheral blood samples before and after patient treatment, we can simultaneously resolve the transcriptional state, clonal lineages, and specificity of TASCs. For instance, identifying gene signatures unique to precursor-depleted T cells responsive to immune checkpoint inhibitors may serve as predictive biomarkers. Furthermore, spatial transcriptomics or multiplex immunofluorescence techniques reveal distinct spatial distributions of TASCs within tumour cores, invasive margins, or tertiary lymphoid structures—a spatial information closely linked to their functional states. Integrating these multidimensional TASCs maps data holds promise for developing artificial intelligence predictive models to guide clinical decision-making.
